# DFFNet: A Dual-Domain Feature Fusion Network for Single Remote Sensing Image Dehazing

**DOI:** 10.3390/s25165125

**Published:** 2025-08-18

**Authors:** Huazhong Jin, Zhang Chen, Zhina Song, Kaimin Sun

**Affiliations:** 1 School of Computer and Information Engineering, Hubei University of Technology, Wuhan 430068, China; jinhuazhong@hbut.edu.cn (H.J.); 102311289@hbut.edu.cn (Z.C.); 2 Hubei Provincial Key Laboratory of Green Intelligent Computing Power Network, Wuhan 430068, China; 3 State Key Laboratory of Information Engineering in Surveying, Mapping and Remote Sensing, Wuhan University, Wuhan 430010, China; sunkm@whu.edu.cn

**Keywords:** remote sensing image dehazing, feature fusion, frequency-spatial modeling

## Abstract

Single remote sensing image dehazing aims to eliminate atmospheric scattering effects without auxiliary information. It serves as a crucial preprocessing step for enhancing the performance of downstream tasks in remote sensing images. Conventional approaches often struggle to balance haze removal and detail restoration under non-uniform haze distributions. To address this issue, we propose a Dual-domain Feature Fusion Network (DFFNet) for remote sensing image dehazing. DFFNet consists of two specialized units: the Frequency Restore Unit (FRU) and the Context Extract Unit (CEU). As haze primarily manifests as low-frequency energy in the frequency domain, the FRU effectively suppresses haze across the entire image by adaptively modulating low-frequency amplitudes. Meanwhile, to reconstruct details attenuated due to dense haze occlusion, we introduce the CEU. This unit extracts multi-scale spatial features to capture contextual information, providing structural guidance for detail reconstruction. Furthermore, we introduce the Dual-Domain Feature Fusion Module (DDFFM) to establish dependencies between features from FRU and CEU via a designed attention mechanism. This leverages spatial contextual information to guide detail reconstruction during frequency domain haze removal. Experiments on the StateHaze1k, RICE and RRSHID datasets demonstrate that DFFNet achieves competitive performance in both visual quality and quantitative metrics.

## 1. Introduction

With the rapid advancement of remote sensing technology, remote sensing images have been widely applied in numerous fields such as civil engineering, forestry resource surveys, and geological exploration [1,2,3,4,5]. However, these images are frequently degraded by environmental factors such as atmospheric scattering and cloud, leading to issues such as excessive whitening, reduced contrast, and obscured content [6]. Such degradations severely undermine the utility of remote sensing images in downstream tasks. Consequently, image dehazing has become an essential preprocessing step to enhance the quality and reliability of remote sensing applications.

Early remote sensing image dehazing methods were typically grounded in the atmospheric scattering model [7], where I(x) represents the observed hazy image at pixel *x*, and the model is expressed as

(1) I(x)=J(x)·t(x)+A·(1−t(x)).

In this model, the global atmospheric light *A* and the transmission map t(x) are estimated using handcrafted priors, and the haze-free image J(x) is subsequently reconstructed. Representative priors include the Dark Channel Prior (DCP) [8], Color Attenuation Prior (CAP) [9], and the haze-line model [10]. Fusion-based approaches [11] have also been explored to enhance contrast via multi-scale information. While effective in certain scenarios, these methods rely heavily on assumptions that limit their generalizability in complex haze conditions.

In contrast, deep learning-based approaches have shown superior feature extraction and modeling capabilities, enabling more robust dehazing in complex scenes. DehazeNet [12] and AOD-Net [13] were among the first to introduce convolutional neural networks (CNNs) to this task, demonstrating notable improvements in modeling capacity. Subsequent works such as GridDehazeNet [14] and KFA-Net [15] further improved performance through multi-scale fusion and attention mechanisms, enhancing fine detail recovery. To enhance perceptual quality, generative adversarial networks (GANs), including FD-GAN [16] and recent work by Shen et al. [17], have employed adversarial learning to generate more natural textures. However, GAN-based models often suffer from training instability. Recently, Transformer-based architectures have attracted attention due to their strong long-range dependency modeling capabilities. Methods such as DehazeFormer [18] and GTMNet [19] have demonstrated impressive results, particularly in remote sensing scenarios. Nonetheless, they often struggle with preserving local structures and impose high computational costs. In addition, diffusion models have emerged as a promising direction owing to their stability and generative fidelity. For instance, Huang et al. [20] pioneered the use of diffusion models for dehazing. Yet, their iterative sampling process remains time-consuming.

Despite the significant progress in image dehazing in recent years, most existing approaches still rely heavily on spatial domain modeling while overlooking the potential of frequency domain information. In fact, frequency domain processing offers notable advantages for haze suppression [21,22,23]: since the haze-related signals is primarily concentrated in the low-frequency amplitude components, modulating these components helps to suppress haze more effectively. In contrast, spatial domain methods exhibit strong sensitivity to local details, making them more suitable for restoring details. Therefore, establishing an effective synergy between spatial and frequency domains features holds promise for more precise dehazing. Building on these insights, we design an end-to-end dehazing network via dual-domain feature fusion for remote sensing image. The contributions of this paper are as follows:

- We propose a dual-domain parallel modeling module, termed the Spatial–Frequency Block (Spa-Fre Block), which incorporates a spatial branch using Context Extract Unit (CEU) to capture multi-scale contextual features for local details restruction and a frequency branch using Frequency Restore Unit (FRU) to modulate amplitude for global haze suppression.
- We introduce a Dual-Domain Feature Fusion Module (DDFFM), which serves as a feature fusion attention mechanism. This module builds contextual dependencies between spatial and frequency features based on the self-attention mechanism. To address the limited local modeling capability of Transformers, a parallel convolutional branch is introduced to enhance local perception. In addition, a fusion weight modulation mechanism is employed to adaptively balance spatial and frequency features across different stages.
- We propose an end-to-end network, DFFNet, which features a dual-domain fusion architecture. It incorporates skip connections and attention mechanisms to enhance feature representation, while leveraging the atmospheric scattering model to impose explicit constraints on the dehazing process.

## 2. Related Work

### 2.1. Image Dehazing

Image dehazing, a fundamental problem in the field of image enhancement, was initially addressed using contrast adjustment techniques and physical prior-based models.

Enhancement-based methods, such as histogram equalization [24], Retinex [25], MSRCR [26,27], and homomorphic filtering [28], restore images by improving contrast or separating reflectance components. Physical prior-based methods rely on the atmospheric scattering model in Equation (1) to estimate parameters like the transmission map t(x) and atmospheric light *A* through scene statistics. Representative works include Dark Channel Prior (DCP) [8], which assumes low intensity in non-sky regions; Non-Local Image Dehazing [10], leveraging repetitive color patterns; TAN [29], maximizing local contrast; and Fattal’s method [30], based on independent component analysis of reflectance. While these approaches perform well under their respective assumptions, their heavy reliance on such priors—e.g., DCP depends on non-sky regions—limits their effectiveness and generalization in scenarios involving white objects, sky regions, or complex illumination.

With the advancement of deep learning, learning-based dehazing methods have gained prominence in image dehazing. One approach integrates deep networks with physical models to estimate key parameters. For example, DehazeNet [12] employs multi-layer CNNs to extract multi-scale features and uses bilateral ReLU activation for transmission estimation. MSCNN [31] performs coarse-to-fine multi-scale transmission estimation to guide dehazing. AOD-Net [13] consolidates transmission and atmospheric light into a single parameter K(x), mitigating error accumulation. DMMFD [32] combines classical atmospheric scattering model decomposition with deep learning through a multi-model fusion framework. Alternatively, other methods focus on optimizing network architectures, loss functions, and similar components. DCPDN [33] utilizes densely connected pyramid networks to enhance multi-scale feature usage; CycleGAN [34] employs cycle-consistency loss for unpaired haze-to-clear image translation; FD-GAN [35] introduces a fused discriminator that leverages both low- and high-frequency features to guide the generator, exploring more effective end-to-end dehazing.

In recent years, Transformer models have demonstrated strong capabilities in modeling long-range dependencies and capturing global context, leading to notable improvements in image dehazing performance. For instance, DehazeFormer [18] leverages a Swin Transformer backbone with an enhanced window attention mechanism to capture spatial context. AIDTransformer [36] incorporates deformable multi-head attention and spatial attention offset modules, combined with edge-enhanced skip connections, to focus on relevant contextual information while preserving texture and edge details. PCSformer [37] introduces sliding cross-shaped stripes and local–global proxy Transformer blocks to strengthen contextual interactions. Additionally, some approaches have explored the joint utilization of spatial and frequency domain features for dehazing. FOTformer [22] employs frequency-aware attention and content reconstruction networks in the frequency domain, effectively integrating spatial and frequency features to improve the recovery of complex textures and structures. SFAN [23] utilizes hybrid expert modulation and frequency decoupling learning to dynamically adapt to multi-scale features, efficiently handling frequency domain information.

Despite significant progress in image dehazing and some recent works have incorporated frequency domain processing to enhance global modeling, most existing methods perform spatial–frequency feature fusion either sequentially or via channel concatenation, without adequately accounting for their inherent differences. This often leads to feature redundancy or ineffective fusion. Therefore, designing more effective spatial–frequency fusion strategies is critical to fully leverage the complementary strengths of both domains and holds promise for improving dehazing performance in complex haze conditions.

### 2.2. Feature Fusion

Feature fusion is widely applied in image processing, aiming to integrate multi-level, multi-scale, or multi-modal features to enhance the network’s representational capacity.

Early feature fusion methods primarily relied on multi-scale architectures to extract both local textures and global contours through varying receptive fields, followed by simple concatenation or element-wise addition to integrate features. For instance, MSD-Net [38] employs multi-branch dilated convolutions to capture multi-scale representations, combined with an adaptive fusion strategy to enable cross-scale complementarity—effectively restoring object structures and haze boundaries. Similarly, the U-Net architecture [39] leverages skip connections between the encoder and decoder to fuse shallow detail features with deeper semantic information, making it a widely adopted framework in remote sensing image dehazing. However, such straightforward fusion strategies often fail to distinguish the relative importance of features across different regions, which can lead to redundant or irrelevant information interfering with the network’s representational capacity.

To enhance fusion quality, attention mechanisms have been widely adopted as adaptive weighting strategies in feature fusion. For example, FFA-Net [40] incorporates channel and pixel attention mechanisms at each layer to effectively highlight key regions for dehazing and improve overall reconstruction quality. MSF^2^DN [41] achieves extensive multi-scale feature interaction through multi-stream fusion modules and dense connections, demonstrating superior performance on real haze images. PSPAN [42] leverages pyramid spatial weighted pixel attention to build multi-scale attention pathways and further boosts cross-scale feature integration efficiency via a feature aggregation module.

With the growing application of Transformers in low-level vision tasks, integrating CNNs and Transformers has emerged as a promising direction for feature fusion [43,44]. This hybrid approach leverages the strengths of CNNs in capturing local details and the capability of Transformers to model long-range dependencies, balancing both local textures and global structures. For instance, ref. [45] employs a Transformer branch to model long-range dependencies and incorporates a cross-attention mechanism to facilitate effective feature interaction between CNN and Transformer representations, resulting in more refined image details and a more coherent global structure. DEA-Net [46] further enhances this synergy by introducing a content-guided attention mechanism in its detail enhancement module, generating adaptive spatial weight maps for each channel to enable differentiated fusion across feature channels.

Against this background, we propose an enhanced self-attention fusion mechanism that leverages the complementary strengths of CNNs and Transformers. This mechanism computes self-attention across features extracted from both the spatial and frequency branches, allowing the model to capture contextual dependencies while simultaneously employing a dedicated convolutional branch to emphasize local structure. To address the high computational cost typically associated with Transformer-based attention, we further reformulate the Query–Key multiplication as an element-wise operation in the frequency domain. This transformation preserves the representational capacity of self-attention while significantly reducing spatial complexity, thereby improving the overall efficiency of the network.

## 3. Method

### 3.1. Overall Framework

**Figure 1:**
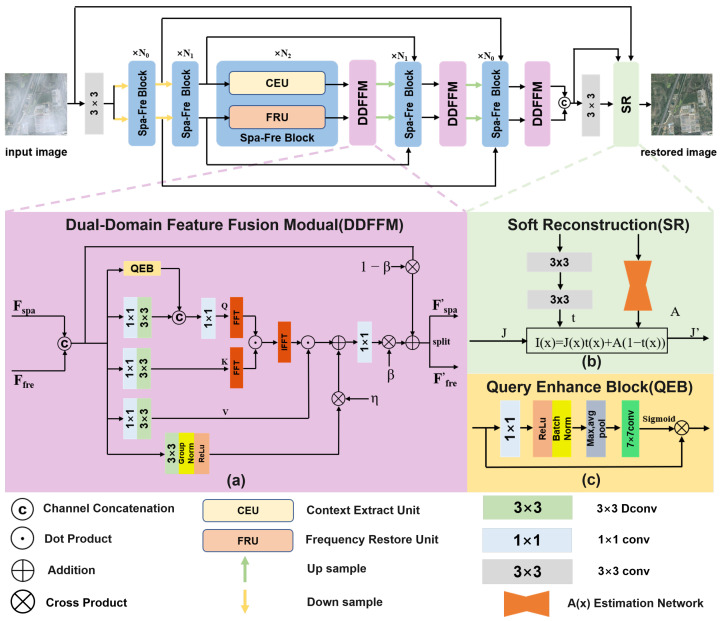
The overall network architecture of DFFNet (**top**). The middle parts illustrate (**a**) the Dual-Domain Feature Fusion Module (DDFFM), (**b**) the implementation details of soft reconstruction, and (**c**) the Query Enhance Block (QEB) within the DDFFM. The **bottom** part contains the symbol legend for basic operations such as convolution and concatenation.

As illustrated in Figure 1, DFFNet adopts a typical encoder–decoder architecture. During encoding, the network progressively downsamples the input to compress spatial information and increase the number of feature channels. Meanwhile, skip connections from both the spatial and frequency domains are preserved separately for later decoding stage. The decoder mirrors the structure of the encoder and gradually restores the spatial resolution of the image. At each decoding layer, the current feature is fused with the corresponding same-domain skip feature via SKFusion. Notably, DFFNet delays the interaction between spatial and frequency domain features until the decoding phase, thereby minimizing interference between early-stage features from distinct domains. This delayed fusion is later implemented via the DDFFM. Finally, a soft reconstruction module, inspired by [47], is employed to constrain the haze distribution and generate the final dehazed image.

### 3.2. Spa-Fre Block

**Figure 2:**
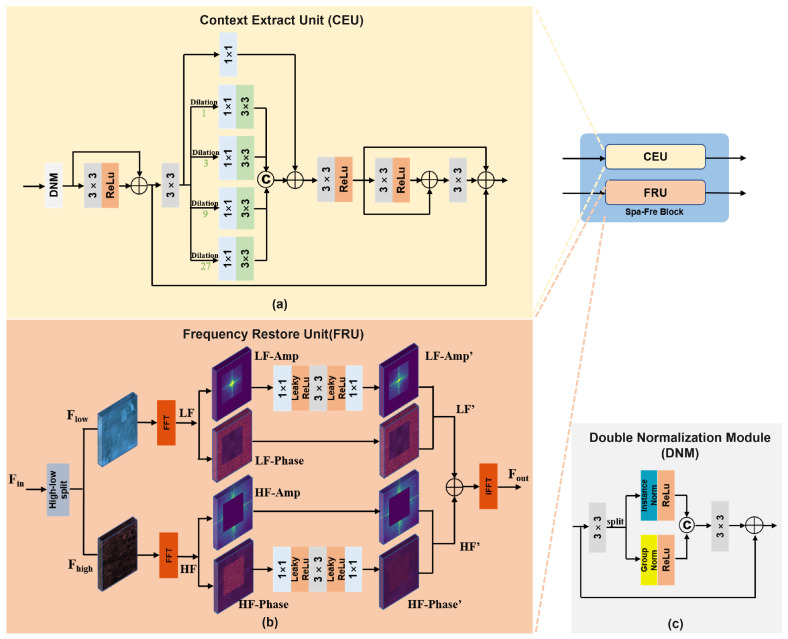
Architecture of the Spa-Fre Block. The subfigures illustrate (**a**) the Context Extract Unit (CEU), (**b**) the Frequency Restore Unit (FRU) and (**c**) the Double Normalization Module.

The Spa-Fre Block is one of the core functional components of DFFNet. It consists of two parallel branches, the spatial domain unit (CEU) and the frequency domain unit (FRU) as illustrated in Figure 2a and Figure 2b, respectively. This parallel design facilitates the independent processing of spatial and frequency features within the same layer, thereby reducing interference caused by serial computation. The detailed workflow of the Spa-Fre Block is as follows.

Given spatial and frequency features $F_{\mathrm{spa}},F_{\mathrm{fre}}\in R^{B\times C\times H\times W}$ from the previous layer (where *B* is batch size, *C* is channels, and *H* and *W* are height and width), these are separately fed into the CEU and FRU for further processing. The operation can be formulated as

(2) $$\mathrm{Spa-FreBlock}\left(F_{\mathrm{spa}},F_{\mathrm{fre}}\right)=\left\{\mathrm{CEU}\left(F_{\mathrm{spa}}\right),\mathrm{FRU}\left(F_{\mathrm{fre}}\right)\right\}$$

**(a) FRU.** In the FRU, we adopt the adaptive threshold mask *M* proposed in [23] to separate the input feature $F_{in}$ into high-frequency ($F_{high}$) and low-frequency ($F_{low}$) components as follows:

(3) $$F_{low}=F_{\mathrm{in}}⊙M,\;F_{high}=F_{\mathrm{in}}⊙\left(1-M\right)$$

where ⊙ denotes element-wise multiplication. To further process frequency-specific information, we transform both components into the frequency domain via the Fast Fourier Transform (FFT):

(4) $$LF=FFT\left(F_{low}\right),\;HF=FFT\left(F_{high}\right)$$

Next, to suppress haze-related information, we modulate the amplitude $A_{\mathrm{low}}$ of the low-frequency component LF using a series of convolutional and LeakyReLU layers:

(5) $$A_{\mathrm{low}}^{\prime }={\mathrm{Conv}}_{1\times 1}\left(\sigma \left({\mathrm{Conv}}_{3\times 3}\left(\sigma \left({\mathrm{Conv}}_{1\times 1}\left(A_{\mathrm{low}}\right)\right)\right)\right)\right)$$

where σ is the activation function LeakyReLU. The modulated low-frequency feature is then denoted as $LF^{\prime }$. For the high-frequency component HF, we focus on phase enhancement to strengthen global structural cues. The phase component $P_{\mathrm{high}}$ is modulated as

(6) $$P_{\mathrm{high}}^{\prime }={\mathrm{Conv}}_{1\times 1}\left(\sigma \left({\mathrm{Conv}}_{3\times 3}\left(\sigma \left({\mathrm{Conv}}_{1\times 1}\left(P_{\mathrm{high}}\right)\right)\right)\right)\right)$$

This results in the refined high-frequency feature $HF^{\prime }$. Finally, the restored frequency domain representation is reconstructed by applying an inverse FFT:

(7) $$F_{\mathrm{out}}=\mathrm{IFFT}2\left(LF^{\prime }+HF^{\prime }\right)$$

**(b) CEU.** Inspired by the work in [48], the CEU employs multiple dilated convolutions with different dilation rates to capture multi-scale receptive fields. Before performing detail extraction, we introduce a Double Normalization Module (DNM) (Figure 2c) to enhance feature representation and stabilize training.

In the DNM, the input spatial feature $F_{\mathrm{spa}}$ is first processed by a 3×3 convolution and then evenly split along the channel dimension into two parts, $F_{\mathrm{spa}}^{1}$ and $F_{\mathrm{spa}}^{2}$:

(8) $$F_{\mathrm{spa}}^{1},F_{\mathrm{spa}}^{2}=\mathrm{Split}\left({\mathrm{Conv}}_{3\times 3}\left(F_{\mathrm{spa}}\right)\right)$$

Each part is then normalized using Instance Normalization and Group Normalization, respectively, followed by ReLU activation:

(9) $$F_{\mathrm{spa}}^{\prime 1},F_{\mathrm{spa}}^{\prime 2}=\sigma \left(N_{\mathrm{ins}}\left(F_{\mathrm{spa}}^{1}\right)\right),\;\sigma \left(N_{\mathrm{group}}\left(F_{\mathrm{spa}}^{2}\right)\right)$$

The two normalized features are concatenated along the channel axis and passed through another 3×3 convolution, followed by a residual connection with the original input:

(10) $$F_{\mathrm{spa}}^{\mathrm{DNM}}={\mathrm{Conv}}_{3\times 3}\left(\mathrm{Concat}\left(F_{\mathrm{spa}}^{\prime 1},F_{\mathrm{spa}}^{\prime 2}\right)\right)+F_{\mathrm{spa}}$$

After DNM, the feature is enhanced via a 3×3 convolution followed by ReLU, and added to the residual $F^{\mathrm{DNM}}$ to produce the refined feature $F_{\mathrm{enh}}$:

(11) $$F_{\mathrm{enh}}=\mathrm{ReLU}\left({\mathrm{Conv}}_{3\times 3}\left(F_{\mathrm{spa}}^{\mathrm{DNM}}\right)\right)+F_{\mathrm{spa}}^{\mathrm{DNM}}$$

Then, a 3×3 convolution is applied, followed by a series of parallel dilated convolutions, where each branch is first processed by a 1×1 convolution:

(12) $$D_{i}={\mathrm{DiatConv}}_{3\times 3,r=i}\left({\mathrm{Conv}}_{1\times 1}\left(F_{\mathrm{enh}}\right)\right),\;i\in \left\{1,3,9,27\right\}$$

The resulting multi-scale features are aggregated via channel-wise concatenation and a residual identity connection:

(13) $$F_{\mathrm{multi}}=\mathrm{Concat}\left({\left\{D_{i}\right\}}_{i\in \{1,3,9,27\}}\right)+\mathrm{Identity}\left(F_{\mathrm{conv}}\right)$$

Finally, a series of 3×3 convolutions with ReLU activations is applied to progressively refine the features, producing the final spatial domain output $F_{\mathrm{spa}}^{\prime }$.

### 3.3. DDFFM

DDFFM is designed to integrate spatial and frequency domain features. By jointly modeling global dependencies and local structural cues, DDFFM facilitates a more refined fusion to handle spatially non-uniform haze more effectively. It works as follows:

**(1) Joint Feature Construction.** Given spatial and frequency domain features $F_{\mathrm{spa}},F_{\mathrm{fre}}$, we first concatenate them along the channel dimension to form a unified representation:

(14) $$F=\mathrm{Concat}(F_{\mathrm{spa}},F_{\mathrm{fre}})$$

**(2) Global Dependency Modeling.** The unified feature *F* is passed through a 1×1 convolution followed by a 3×3 depthwise separable convolution to produce the query (*Q*), key (*K*), and value (*V*) features:

(15) $$F^{\prime }={\mathrm{Conv}}_{1\times 1}\left(F\right)$$

(16) $$Q,K,V={\mathrm{Conv}}_{\mathrm{DW},3\times 3}\left(F^{\prime }\right)$$

To enhance the expressiveness of the query feature *Q*, we incorporate a Query Enhance Block (QEB). QEB begins with a 1×1 convolution followed by ReLU activation and batch normalization. It then extracts statistical priors via max and average pooling, applies a 7×7 convolution for modulation, and generates attention weights through a sigmoid activation to produce the enhanced query

(17) $$Q_{\mathrm{enh}}=\mathrm{QEB}\left(Q\right)$$

Next, $Q_{\mathrm{enh}}$ and *K* are partitioned into non-overlapping P×P blocks and transformed into the frequency domain using FFT, yielding $Q_{\mathrm{fft}}$ and $K_{\mathrm{fft}}$. Dot-product attention is computed in the frequency domain to reduce spatial complexity:

(18) $$Z_{\mathrm{fft}}=Q_{\mathrm{fft}}⊙K_{\mathrm{fft}}$$

The resulting features are converted back to the spatial domain via inverse FFT, and layer normalization is applied to produce $Z_{\mathrm{norm}}$. Finally, weighted aggregation of the Value features *V* is then performed using matrix multiplication:

(19) $$Y_{G}=Z_{\mathrm{norm}}\cdot V$$

**(3) Local Detail Enhancement.** To capture local structure, the original joint feature *F* is processed using 3×3 convolutions, Group Normalization, and ReLU activation, yielding local feature $Y_{L}$:

(20) $$Y_{L}=\mathrm{ReLU}\left(\mathrm{GroupNorm}\left({\mathrm{Conv}}_{3\times 3}\left(F\right)\right)\right)$$

We design a learnable parameter η to balance the global and local contributions:

(21) $$Y={\mathrm{Conv}}_{1\times 1}\left(Y_{G}+\eta \cdot Y_{L}\right)$$

**(4) Fusion Control.** Following the findings in [49,50], which suggest cross-domain fusion in later stages improves performance, we introduce a learnable parameter β to adjust the contribution of fusion:

(22) Y=Y·β+F·(1−β)

**(5) Feature Redistribution.** Finally, the fused feature *Y* is split along the channel dimension to generate the enhanced spatial and frequency outputs, $F_{\mathrm{spa}}^{\prime }$ and $F_{\mathrm{fre}}^{\prime }$, respectively,

(23) $$F_{\mathrm{spa}}^{\prime },F_{\mathrm{fre}}^{\prime }=\mathrm{Split}\left(Y\right)$$

### 3.4. Loss Function

To jointly optimize both local detail preservation and global semantic consistency, we design a composite loss function comprising pixel-wise L1 loss, perceptual loss, frequency domain loss, and a reconstruction consistency loss for soft dehazing.

Perceptual loss is employed to capture high-level semantic similarity between the predicted image *p* and the ground truth *g*. It leverages multi-level features extracted from a pretrained VGG-19 network, specifically from layers conv1_2 to conv5_2:

(24) $$L_{\mathrm{perceptual}}=\sum_{i=1}^{5}\omega_{i}{∥F^{i}\left(g\right)-F^{i}\left(p\right)∥}_{1}$$

where $\omega_{i}$ is the weight for the *i*-th layer $F^{i}\left(\cdot \right)$.

Pixel-level L1 loss is used to ensure point-wise fidelity between predictions and ground truth:

(25) $$L_{L1}={∥g-p∥}_{1}$$

For frequency loss, it is adopted following [51] to emphasize consistency in the spectral domain. Both predicted and ground truth images are transformed via FFT, and the difference is measured with adaptive weighting:

(26) $$L_{\mathrm{FFL}}=\frac{1}{MN}\sum_{u=0}^{M-1}\sum_{v=0}^{N-1}w\left(u,v\right){\left|F_{r}\left(u,v\right)-F_{f}\left(u,v\right)\right|}^{2}$$

where the dynamic weight w(u,v) is defined as

(27) $$w\left(u,v\right)={\left|F_{r}\left(u,v\right)-F_{f}\left(u,v\right)\right|}^{\alpha }$$

Here, $F_{r}$ and $F_{f}$ represent the frequency components of the reference and predicted images, respectively. The exponent α controls the sensitivity to frequency differences and is set to 1 in our implementation.

Soft reconstruction loss is introduced to improve the estimation accuracy of the transmission map A(x) and the hazy image I(x). It minimizes the L1 distance between the input hazy image *h* and the re-synthesized haze image *I*:

(28) $$L_{\mathrm{haze}-L1}={∥I-h∥}_{1}$$

Finally, the overall loss function is defined as a weighted sum:

(29) $$L_{\mathrm{total}}=\lambda_{1}L_{L1}+\lambda_{2}L_{\mathrm{perceptual}}+\lambda_{3}L_{\mathrm{FFL}}+\lambda_{4}L_{\mathrm{haze}-L1}$$

In this paper, the weights are set as $\lambda_{1}=\lambda_{2}=\lambda_{3}=1$ and $\lambda_{4}=0.1$.

## 4. Experiments

### 4.1. Datasets

We evaluate our proposed DFFNet on three publicly available haze remote sensing datasets: StateHaze1k [52], RICE [53], and RRSHID [54]. The image resolution of StateHaze1k and RICE is 512×512, whereas that of RRSHID is 256×256.

**StateHaze1k** contains three subsets with different levels of haze density, namely haze1k_thin, haze1k_moderate, and haze1k_thick. Each subset includes approximately 400 pairs of synthesized RGB remote sensing images; among them, 320 image pairs are used for training, 45 for testing, and 35 for validation.

In the haze1k_thick subset, most images are dominated by dense haze with relatively concentrated distribution. The haze1k_thin subset contains images with light haze, where some regions are nearly haze-free. In contrast, the haze1k_moderate subset exhibits the most complex haze distribution, featuring a mix of thin haze, dense haze, and clear areas, thus demonstrating significant non-uniformity.

**RICE** is a real-world dataset created by Google Earth for the task of remote sensing image dehazing. It is divided into two subsets: RICE1 and RICE2.

In RICE1, most images have evenly distributed haze, with only about 10 images showing uneven haze patterns. To ensure representative coverage of such characteristics, we allocate 5 uneven haze images to both the training and testing sets. In total, the training set consists of 402 image pairs, and the testing set includes 98 pairs.

RICE2 contains images of coastal and inland scenes obscured by real clouds. After manual selection, 24 representative images are designated for the testing set. In total, this subset contains 590 images for training and 146 for testing.

**RRSHID** is a large-scale real-world dataset featuring paired hazy and haze-free remote sensing images. Unlike synthetic datasets that rely on simplified atmospheric models, RRSHID captures authentic atmospheric phenomena, such as heterogeneous haze densities and spatial distributions within individual images, intricate interactions between haze and diverse land cover types, and color deviations caused by variations in natural lighting and atmospheric composition. This makes it well-suited for validating the robustness of models in real-world scenarios. It is stratified by haze density into three subsets: RRSHID_thin, RRSHID_moderate, and RRSHID_thick.

The RRSHID_thin subset includes 763 image pairs, with 610 for training, 76 for testing, and 77 for validation. The RRSHID_moderate subset, the largest among the three, contains 1526 pairs, allocated as 1220 for training, 152 for testing, and 154 for validation. The RRSHID_thick subset has 764 pairs, with 611 for training, 76 for testing, and 77 for validation.

### 4.2. Evaluation Metrics

We evaluate the quality of restored images using three commonly adopted standard metrics, which have been widely used in prior works (e.g., [37,55,56]): Peak Signal-to-Noise Ratio (PSNR), Structural Similarity Index (SSIM), and Learned Perceptual Image Patch Similarity (LPIPS). These metrics collectively assess the restoration performance from pixel-level, structural, and perceptual perspectives.

PSNR quantifies the pixel-wise error between the restored and reference images. Higher PSNR values indicate lower distortion and better fidelity. SSIM measures perceptual similarity by comparing luminance, contrast, and structural information; values closer to 1 denote higher structural consistency. LPIPS evaluates perceptual similarity based on deep features extracted from a pretrained neural network, capturing high-level semantic differences. Lower LPIPS scores correspond to better perceptual alignment.

Together, these metrics provide a comprehensive evaluation of both global semantics and local detail preservation after dehazing. The formulas for each metric are as follows:

(30) $$\mathrm{PSNR}=10\cdot \log_{10}\left(\frac{\max_{I}^{2}}{\mathrm{MSE}}\right),\;\mathrm{MSE}=\frac{1}{h\cdot w}\sum_{i=0}^{h-1}\sum_{j=0}^{w-1}{\left(x\left(i,j\right)-y\left(i,j\right)\right)}^{2}$$

where $\max_{I}$ is the maximum pixel value in the image, $\log_{10}$ is base-10 logarithm, *x* is the dehazed image produced by the model, *y* is the ground truth, and h,w are the height and width of the image, respectively:

(31) $$\mathrm{SSIM}\left(x,y\right)=\frac{\left(2\mu_{x}\mu_{y}+C_{1}\right)\left(2\sigma_{xy}+C_{2}\right)}{\left(\mu_{x}^{2}+\mu_{y}^{2}+C_{1}\right)\left(\sigma_{x}^{2}+\sigma_{y}^{2}+C_{2}\right)}$$

where μ denotes the mean, $\sigma^{2}$ is the variance, $\sigma_{xy}$ is the covariance of *x* and *y*, and $C_{1}$ and $C_{2}$ are constants used to prevent division by zero:

(32) $$\mathrm{LPIPS}\left(x,y\right)=\sum_{l}w_{l}\cdot {∥{\hat{f}}_{l}\left(x\right)-{\hat{f}}_{l}\left(y\right)∥}_{2}^{2}$$

where ${\hat{f}}_{l}\left(x\right)$ and ${\hat{f}}_{l}\left(y\right)$ represent the feature maps of images *x* and *y* extracted at the *l*-th layer of a pretrained network, and $w_{l}$ is the weight coefficient of that layer. In this paper, AlexNet is used as the backbone network.

### 4.3. Implementation Details

We implement and train DFFNet using the PyTorch framework with an NVIDIA RTX 4090 GPU (24 GB). During training, RGB remote sensing images are randomly cropped to a size of 256×256, with a batch size of 4. During validation, evaluation is conducted on full-resolution images without cropping. On each subset, DFFNet is trained for 500 epochs, with the total training time being approximately 5–7 h. This time includes per-epoch validation on the training set, which can vary across datasets depending on their size.

In DFFNet, the number of Spa-Fre Blocks in each stage is set to $\left[N_{0},N_{1},N_{2}\right]=\left[1,1,2\right]$, and the corresponding embedding channel dimensions are [24,48,96]. We use the Adam optimizer with an initial learning rate of $1.0\times 10^{-4}$, $\beta_{1}=0.9$, $\beta_{2}=0.999$, $\epsilon =1.0\times 10^{-8}$, and without weight decay. The learning rate is adjusted using a cosine annealing schedule with $T_{\max}=500$ (i.e., the total number of training epochs) and a minimum learning rate $\eta_{\min}=1.0\times 10^{-6}$.

### 4.4. Experimental Results and Analysis

We compare DFFNet with classical dehazing methods and several recent high-impact approaches. These include the prior-based DCP [8], the CNN-based AOD-Net [13], FFA-Net [40], and DCMPNet [57], the Transformer-based DehazeFormer [18], SFAN [23], and the diffusion model-based RSHazeDiff [58]. It is worth noting that SFAN also incorporates frequency domain processing, which aligns it conceptually with our approach and makes the comparison more relevant. To ensure a fair comparison, we adopt the same batch size and input resolution as used in DFFNet, while keeping all other settings consistent with the original implementations of the respective methods. The experimental results are presented as follows.

**(1) Results on StateHaze1K:** The quantitative evaluation results of different methods on the StateHaze1K dataset are shown in Table 1, which shows that our method achieves top or second-best performance across all haze levels on the StateHaze1K dataset. Notably, it ranks first in both PSNR and LPIPS in light and medium haze conditions, and maintains competitive results under heavy haze, reflecting its effectiveness in both perceptual quality and robustness under varying atmospheric degradations. The “Average” value reported in the table is computed as the arithmetic mean across all haze-level subsets, consistent with the standard practice in related works.

**Figure 3:**
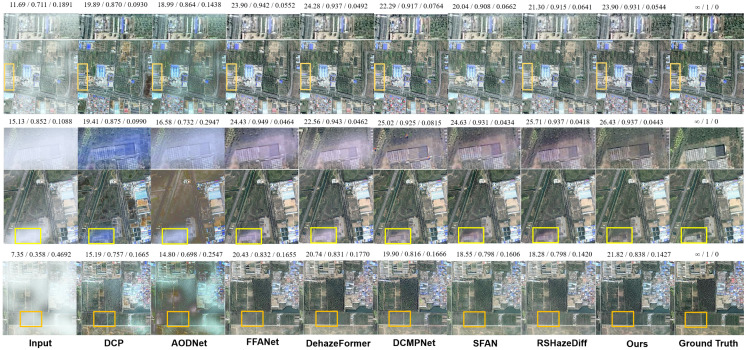
Qualitative comparison on the StateHaze1K dataset. Images in the first, second, and third rows are sampled from the **haze1k_thin**, **haze1k_moderate**, and **haze1k_thick** subsets, respectively. The numbers above each image indicate PSNR, SSIM, and LPIPS scores.

The qualitative visual results (see Figure 3) further support these conclusions. In light haze images, DCP exhibits noticeable color oversaturation, while AOD-Net suffers from color distortion and large areas of residual haze. Although FFA-Net, SFAN, and RSHazeDiff perform relatively well in general, there are still slight haze remnants along the left edge of the image. In contrast, both DehazeFormer and our method produce cleaner, more natural-looking images with better detail restoration and perceptual consistency.

In the medium haze scene, the input image contains haze that is unevenly distributed and varies in thickness. Except for our method, most others show varying degrees of residual haze in the denser lower part of the image. DCP, AOD-Net, and FFA-Net show obvious remnants with a bluish tint, while DCMPNet, SFAN, and RSHazeDiff leave only small residual areas but still show whitening artifacts. Our method, however, achieves complete haze removal and maintains an overall tone closer to the ground truth, aligning well with human visual perception.

In heavy haze images, although most methods can remove the majority of haze, some, such as DCMPNet and RSHazeDiff, still show whitening in grassy areas. Our method effectively restores true color in these regions, maintaining consistency with the surrounding context and demonstrating its precise modeling of contextual information in strongly degraded regions.

**Figure 4:**
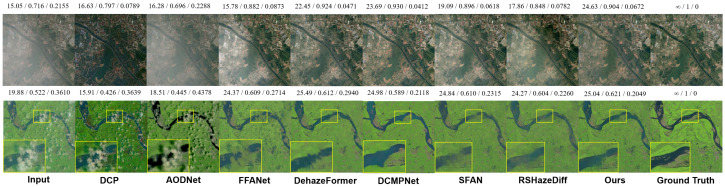
Qualitative comparison on the RICE dataset. Images in the first and second rows are sampled from the **RICE1** and **RICE2** subsets, respectively. The values displayed above each image indicate PSNR, SSIM, and LPIPS metrics.

**(2) Results on RICE:** As shown in Table 2, DFFNet achieves the best average performance across PSNR, SSIM, and LPIPS on the RICE dataset. It consistently ranks among the top methods on both RICE1 and RICE2, demonstrating strong capabilities in brightness restoration, structural preservation, and perceptual quality. We also provide qualitative results (Figure 4). In RICE1, we select a lightly hazed image with uneven haze distribution for comparison. Although DCP removes haze globally, it results in severe oversaturation. AOD-Net shows almost no change. While FFA-Net, SFAN, and RSHazeDiff manage to remove the haze, the brightness in the previously hazy areas remains largely unchanged. In contrast, DCMPNet, DehazeFormer, and our DFFNet maintain more uniform brightness across the image, effectively removing haze while preserving natural and consistent colors and contrast. The image shown in RICE2 depicts scenes characterized by light haze and scattered cloud patches. DCP and AOD-Net primarily attempt haze removal by increasing saturation, leaving cloud structures still visible, and AOD-Net incorrectly tints them green. FFA-Net removes the clouds but leaves noticeable green patches in the areas where the clouds were. SFAN and RSHazeDiff show slight improvements but introduce smearing artifacts. By comparison, DCMPNet, DehazeFormer, and our DFFNet not only thoroughly eliminate the haze and clouds but also reasonably restore details in the ground and river regions, delivering a more natural and coherent visual effect.

**Figure 5:**
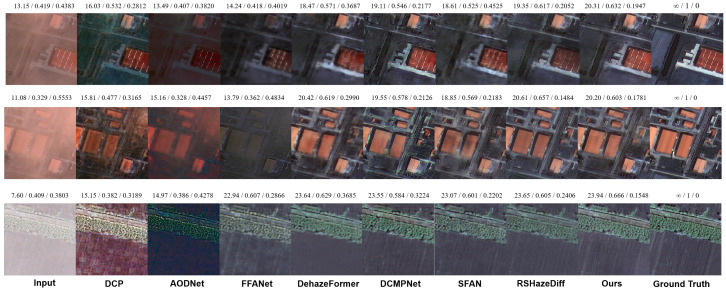
Qualitative comparison on the RRSHID dataset. Images in the first, second, and third rows are sampled from the **RRSHID_thin**, **RRSHID_moderate**, and **RRSHID_thick** subsets, respectively. The numbers above each image indicate PSNR, SSIM, and LPIPS scores.

**(3) Results on RRSHID:** According to the quantitative results presented in Table 3, DFFNet demonstrates superior dehazing performance across all three haze levels as well as in the overall average. It consistently achieves the highest values in PSNR and SSIM, along with the lowest LPIPS, particularly excelling under thick haze conditions. These results indicate that DFFNet possesses strong generalization ability and robustness across a wide range of challenging haze scenarios.It is noteworthy that both DFFNet and SFAN incorporate frequency domain processing mechanisms, yet a clear performance gap exists between the two. Although SFAN maintains relatively high SSIM and low LPIPS under moderate and thick haze, its overall PSNR and average performance are inferior. This can be attributed to the more effective spatial–frequency integration in the architecture of DFFNet. RSHazeDiff also performs competitively across multiple metrics, with particularly strong perceptual quality as reflected by its LPIPS score. However, due to its diffusion-based structure, it incurs significantly higher training and inference costs, which may hinder its deployment in practical applications.The qualitative visual results (Figure 5) demonstrate that our model effectively balances detail restoration and haze removal under all three haze levels. It also shows a relatively accurate recovery of ground object colors, indicating strong visual consistency across varying haze densities.

### 4.5. Efficiency Analysis

#### 4.5.1. Inference Time

We evaluate the runtime efficiency on the Haze1k_thick validation set at the original 512 × 512 resolution with the batch size set to 4. As shown in Table 4, DFFNet requires approximately 0.0997 s per image during inference. While not the fastest, it remains comparable to SFAN (0.0880 s), which also incorporates frequency domain processing. The added cost primarily stems from convolution operations in the spatial domain. Nonetheless, DFFNet achieves a good trade-off between performance and efficiency, with inference time still within an acceptable range.

In contrast, diffusion-based models such as RSHazeDiff are significantly more time-consuming (9.48 s/image) due to their inherently iterative denoising process. For reference, classical methods like DCP, which operate solely on the CPU, are also generally slower than modern CNN-based approaches.

#### 4.5.2. Model Size and Computational Complexity

Table 4 also presents a comparison of model complexity across different dehazing methods in terms of parameter count and FLOPs per 256 × 256 image (except for RSHazeDiff, which operates on a fixed 64 × 64 resolution at the first stage). DFFNet consists of approximately 17.64 million parameters and requires 168.22 GFLOPs, which is higher than lightweight models such as AODNet (0.0018M/0.114G) and frequency-based SFAN (4.03M/16.58G) but still moderate compared to larger networks like FFANet (4.46M/287.53G) and DCMPNet (32.73M/113.63G). RSHazeDiff adopts a two-stage architecture, where the total parameters (109.68M + 0.63M) and FLOPs (15.78G + 6.63G) are reported based on the input sizes specific to each stage.

Although DFFNet achieves a reasonable balance between performance and efficiency, we observe that its relatively high computational complexity mainly stems from the spatial domain branch, particularly the successive 3 × 3 convolutions following dilated operations in each CEU (Table 5). These operations contribute significantly to the overall FLOPs and inference time. Our future work will investigate more lightweight alternatives or convolution-efficient designs to reduce redundancy without sacrificing performance.

#### 4.5.3. Effect of Dilation Rates

In this experiment, we evaluate the performance of five different dilation rate configurations on the *haze1k_moderate* dataset for the image reconstruction task. As shown in Table 6, the configuration (1,3,9,27) achieves the best overall results, with a PSNR of 25.49 dB, SSIM of 0.9066, and the lowest LPIPS (0.0540), indicating superior performance in both structural preservation and perceptual quality. The (1,4,9,16) setting performs slightly worse but still yields competitive results. In contrast, configurations such as (1,2,4,8) and (3,5,7,9) achieve lower reconstruction quality, suggesting that simply using closely spaced dilation rates may limit the model’s receptive field diversity. While larger dilation factors tend to improve performance to some extent, the configuration (1,3,9,36) does not surpass (1,3,9,27), indicating that blindly increasing dilation rates does not always lead to further gains. Excessively large gaps may cause aliasing or the ineffective aggregation of local details. Moreover, higher dilation rates also introduce a marginal increase in inference time. These results demonstrate that the choice of dilation rates significantly impacts model performance. A carefully designed multi-scale spacing is essential for achieving a favorable trade-off between reconstruction quality and computational efficiency.

### 4.6. Ablation Study

#### 4.6.1. Ablation Studies on Model Components

We conduct ablation experiments on the *Haze1k_moderate* dataset to evaluate the contributions of the CEU, FRU, and DDFFM modules, and to examine the effect of arranging CEU and FRU in series. All experiments use identical training settings.

As shown in Table 7, replacing DDFFM with vanilla attention, removing either FRU or CEU, or arranging FRU and CEU in series all lead to noticeable drops in PSNR and other evaluation metrics, compared to the complete DFFNet.

Specifically, replacing DDFFM degrades the effectiveness of spatial–frequency feature fusion, as vanilla attention only models global dependencies within each domain, ignoring local interactions. Removing FRU leads to weaker haze suppression. While FRU still contributes to global haze suppression, removing CEU results in inadequate restoration of local details due to the lack of spatial contextual information.

Moreover, serially connecting FRU and CEU within the same layer degrades performance significantly (PSNR drops by 4.61 dB, SSIM by 0.208). This arrangement introduces redundant noise from FRU that cannot be effectively refined by the subsequent CEU.

**Figure 6:**
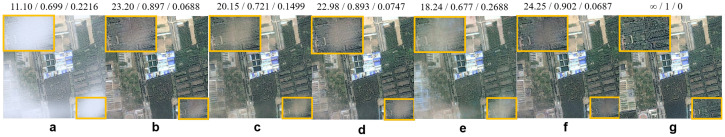
Results of the ablation study. (**a**) Input image. (**b**) CEU + DDFFM. (**c**) CEU + FRU (sequential) + DDFFM. (**d**) CEU + FRU (parallel) + vanilla attention. (**e**) FRU + DDFFM. (**f**) CEU + FRU (parallel) + DDFFM (ours). (**g**) Ground Truth.

Figure 6 provides qualitative evidence that aligns with the analytical insights derived from the ablation results. Compared to variants (b–e), image (f) exhibits the least residual haze in the bottom-right corner and the most visually consistent tone with the surrounding areas, highlighting the superior restoration quality achieved by the complete DFFNet.

#### 4.6.2. Ablation on Learnable Fusion Weights

To evaluate the impact of the two learnable parameters η and β in the proposed DDFFM module on model performance, we conducted an ablation study by comparing the results under different fixed values of these parameters. Specifically, we conducted controlled experiments under four different parameter settings, where both η and β were fixed to either 0.5 or 1. In each setting, all three DDFFM modules in the network used the same values of η and β. The results are summarized in Table 8. Additionally, we recorded the learned values of η and β during standard training, collected from DDFFM modules at different layers, as shown in Table 9.

From the results, we observe that the fixed setting η=0.5,β=0.5 achieves the best performance, yielding the highest PSNR and SSIM as well as the lowest LPIPS. However, the learned values of η and β vary significantly across different layers, suggesting that the model adapts its fusion strategy based on spatial location or semantic content.

#### 4.6.3. Component Effectiveness Analysis

**Figure 7:**
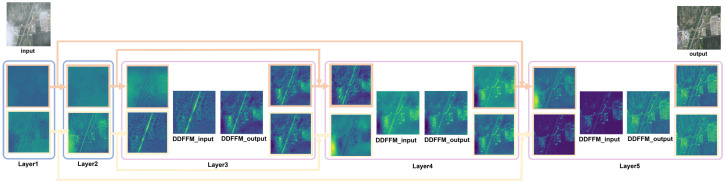
Visualization of PCA-reduced feature maps using the Viridis colormap. Note that in the Viridis colormap, brighter colors indicate higher feature values and do not correspond to the actual image colors.

In this subsection, a representative remote sensing image characterized by non-uniform haze distribution is selected as an input example, where the bottom-left region is heavily obscured by dense haze, while the remaining areas are relatively clear or haze-free. Figure 7 illustrates how the input is progressively processed across different layers of the network. Frequency domain and spatial domain feature maps are highlighted with orange and yellow borders, respectively. Layers 3 to 5 illustrate the feature representations before and after DDFFM fusion. From left to right, the columns correspond to input features from the frequency and spatial branches, concatenated dual-domain features, fused outputs after DDFFM processing, and separately recovered frequency domain and spatial domain outputs obtained via channel-wise decomposition.

In the early feature extraction stages (Layer1–Layer2), the spatial and frequency branches process the input image independently. The spatial domain features preserve texture and structural details with well-defined ground object contours. In contrast, the frequency domain features contain less fine detail but begin to reflect the global distribution of haze across the image. This stage clearly highlights the representational differences between the two branches.

By Layer3, before DDFFM fusion, the frequency domain features begin to exhibit green-colored activations corresponding to hazy regions, indicating that the network has started responding to haze-specific signals. Meanwhile, spatial features remain focused on key structural regions such as roads, retaining stronger local details.

In Layer4, before DDFFM fusion, frequency domain features appear blurred and lack structural sharpness, whereas spatial features maintain rich contextual information due to residual connections from earlier layers (e.g., Layer2). Following fusion via the DDFFM module in Layer4, both branches show evidence of complementary enhancement. Although details in the densest haze regions remain partially suppressed, the overall structural coherence improves.

In Layer5, prior to DDFFM fusion, the frequency domain branch exhibits homogeneous green blobs over the dense haze regions, indicating a strong activation response. Conversely, the spatial branch features are more textured and edge-enhanced, often attributed to skip connections that incorporate earlier feature representations. After final fusion in Layer5, the outputs from both branches become more consistent in color and structure, with significant detail restoration in previously obscured haze regions.

This progression demonstrates the complementary nature of spatial and frequency modeling: while the frequency branch excels at capturing and suppressing global haze artifacts, it is less effective at fine-grained detail recovery. The spatial branch, providing contextual cues, enhances local structural reconstruction.

The proposed DDFFM module enables dynamic feature integration between the two domains, achieving both noise suppression and information enhancement. This mitigates information loss in the frequency branch and reduces interference during fusion, ultimately yielding superior reconstruction quality—particularly in dense haze regions. These observations validate the complementary advantages of dual-domain modeling and highlight the critical role of DDFFM in bridging local detail recovery with global semantic awareness.

## 5. Conclusions

In this study, we proposed DFFNet, an end-to-end dehazing network for remote sensing images that leverages dual-domain feature fusion from spatial and frequency domains. By processing spatial and frequency features in parallel, DFFNet exploits the global modeling capacity of the frequency domain and the fine-grained detail preservation of the spatial domain, effectively addressing the challenges posed by spatially uneven haze. A dedicated fusion module in the decoder selectively integrates complementary features from both domains, while a composite loss function—comprising pixel-wise L1 loss, perceptual loss, frequency domain loss, and soft reconstruction loss to ensure a balanced optimization of local detail and global consistency. Extensive experiments demonstrate that DFFNet achieves competitive performance across various haze conditions and maintains strong generalization ability. While effective, the frequency domain processing still has room for improvement. Our future work will explore more advanced frequency modeling techniques and extend the framework to broader remote sensing image enhancement tasks. Additionally, the spatial branch of the model incurs considerable computational overhead, which poses challenges for deployment. Therefore, we will also investigate more efficient architectural designs to reduce complexity while maintaining performance.

## Figures and Tables

**Table 1:** Quantitative results of different methods on the StateHaze1K dataset. The best and second-best results in each column are **bolded** and underlined, respectively.

Methods	haze1k_thin			haze1k_moderate			haze1k_thick			Average		
	**PSNR**	**SSIM**	**LPIPS**	**PSNR**	**SSIM**	**LPIPS**	**PSNR**	**SSIM**	**LPIPS**	**PSNR**	**SSIM**	**LPIPS**
DCP	19.49	0.868	0.0942	19.71	0.848	0.1065	17.40	0.800	0.1511	18.87	0.839	0.1173
AOD-Net	19.25	0.869	0.1385	17.95	0.798	0.2231	15.64	0.749	0.2300	17.61	0.806	0.1972
FFA-Net	23.75	0.901	0.0738	25.39	0.900	0.0686	21.45	**0.871**	0.1390	23.55	0.890	0.0938
DehazeFormer	23.89	**0.932**	0.0562	25.31	**0.918**	0.0665	21.65	0.868	0.1536	23.62	**0.906**	0.0921
DCMPNet	23.24	0.916	0.0755	23.95	0.888	0.1040	20.84	0.852	0.1443	22.68	0.885	0.1079
SFAN	22.03	0.915	0.0611	24.69	0.894	0.0646	19.71	0.845	0.1399	22.14	0.885	0.0885
RSHazeDiff	22.37	0.915	0.0606	24.85	0.901	0.0601	20.27	0.850	**0.1204**	22.50	0.889	0.0804
DFFNet(Ours)	**23.93**	0.928	**0.0525**	**25.49**	0.906	**0.0540**	**21.87**	0.869	0.1270	**23.76**	0.901	**0.0778**

**Table 2:** Quantitative results of different methods on the RICE dataset. The best and second-best results in each column are **bolded** and underlined, respectively.

Methods	RICE1			RICE2			Average		
	**PSNR**	**SSIM**	**LPIPS**	**PSNR**	**SSIM**	**LPIPS**	**PSNR**	**SSIM**	**LPIPS**
DCP	12.46	0.668	0.2933	22.62	0.598	0.2976	15.63	0.692	0.2955
AOD-Net	18.81	0.819	0.1889	26.48	0.776	0.2226	22.65	0.798	0.2058
FFA-Net	29.16	0.940	0.0354	32.15	0.876	0.0978	30.66	0.908	0.0666
DehazeFormer	29.69	0.946	0.0370	35.27	0.913	0.0827	32.48	0.930	0.0599
DCMPNet	27.65	0.928	0.0510	35.84	0.902	0.0960	31.75	0.915	0.0735
SFAN	29.20	0.944	**0.0347**	35.80	**0.918**	0.0791	32.50	0.931	0.0569
RSHazeDiff	27.99	0.939	0.0437	**35.92**	**0.918**	0.0755	31.96	0.929	0.0596
DFFNet(Ours)	**29.71**	**0.947**	0.0363	35.85	**0.918**	**0.0732**	**32.78**	**0.932**	**0.0550**

**Table 3:** Quantitative results of different methods on the RRSHID dataset. The best and second-best results in each column are **bolded** and underlined, respectively.

Model	RRSHID_thin			RRSHID_moderate			RRSHID_thick			Average		
	**PSNR**	**SSIM**	**LPIPS**	**PSNR**	**SSIM**	**LPIPS**	**PSNR**	**SSIM**	**LPIPS**	**PSNR**	**SSIM**	**LPIPS**
DCP	18.00	0.430	0.2928	15.71	0.412	0.3629	13.92	0.339	0.4178	15.88	0.394	0.358
AODNet	19.18	0.475	0.3356	16.19	0.346	0.4455	17.84	0.437	0.4542	17.74	0.419	0.412
FFANet	21.53	0.562	0.3332	20.88	0.550	0.3527	23.25	0.638	0.3300	21.89	0.583	0.339
DehazeFormer	23.42	0.643	0.3429	23.34	0.623	0.3366	24.27	0.655	0.3588	23.68	0.640	0.346
DCMPNet	23.47	0.638	0.2201	23.14	0.614	0.2178	23.64	0.574	0.3039	23.42	0.609	0.247
SFAN	23.01	0.611	0.3704	22.83	0.601	0.2256	24.26	0.644	0.2292	23.37	0.619	0.275
RSHazeDiff	23.76	0.645	0.1940	**23.74**	0.650	0.2100	24.44	0.668	0.2251	**23.98**	0.654	0.210
DFFNet(Ours)	**23.85**	**0.666**	**0.1859**	23.45	**0.658**	**0.1941**	**24.57**	**0.679**	**0.2137**	23.96	**0.668**	**0.198**

**Table 4:** Comparison of model efficiency in terms of parameter count, FLOPs, and inference time. All FLOPs are computed for 256 × 256 inputs, except for the first stage of RSHazeDiff (computed at 64 × 64 resolution).

Method	Params (M)	FLOPs (G)	Inference Time (s)
DCP	–	–	0.0426
AODNet	0.002	0.114	0.0257
FFANet	4.456	287.533	0.0581
DehazeFormer	1.283	12.056	0.0369
DCMPNet	32.730	113.630	0.0627
SFAN	4.028	16.582	0.0880
RSHazeDiff	110.313	22.413	9.4756
DFFNet(Ours)	17.638	168.223	0.0997

**Table 5:** Effect of different architectural components on model complexity. seq/par refers to whether FRU and CEU process in serial or parallel. **Bold** numbers indicate the complexity of the final model (FRU and CEU in parallel), which is adopted as our default architecture.

FRU	CEU	seq/par	Parameters (M)	FLOPs (G)
✓		–	1.86	15.38
	✓	–	13.70	132.67
✓	✓	seq	14.45	137.25
✓	✓	pal	**17.64**	**168.23**
✓	✓(w/o stacked 3 × 3 convs)	pal	7.18	70.42

**Table 6:** Effect of different dilation rate settings on model performance. **Bold** numbers denote the best performance among all settings, which corresponds to the final chosen configuration.

Dilation Rates	PSNR	SSIM	LPIPS	Time (s/img)
1, 2, 4, 8	25.23	0.9038	0.0593	0.0911
1, 4, 9, 16	25.33	0.9055	0.0595	0.0933
3, 5, 7, 9	25.29	0.9037	0.0603	0.0932
1, 3, 9, 27	**25.49**	**0.9066**	**0.0540**	**0.0944**
1, 3, 9, 36	25.33	0.9051	0.0592	0.0944

**Table 7:** Ablation studies for different components in the model. The best results in each column are **bolded**.

Label	Methods					haze1k_moderate		
	**FRU**	**CEU**	**seq/par**	**DDFFM**	**Vanilla Attention**	**PSNR**	**SSIM**	**LPIPS**
b		✓		✓		25.42	0.888	0.0756
c	✓	✓	seq	✓		20.88	0.698	0.1785
d	✓	✓	par		✓	25.34	0.887	0.0760
e	✓			✓		19.38	0.669	0.3034
f	✓	✓	par	✓		**25.49**	**0.906**	**0.0540**

**Table 8:** Comparison of model performance under different fixed parameters η and β on haze1k_moderate.

η	β	PSNR	SSIM	LPIPS
0.5	0.5	25.35	0.9049	0.0595
1.0	0.5	25.27	0.9039	0.0598
1.0	1.0	25.29	0.9028	0.0602
0.5	1.0	25.27	0.9046	0.0591

**Table 9:** The actual learned parameter values of η and β (for each DDFFM module) by the model on Haze1k_moderate.

Module	η	β	PSNR	SSIM	LPIPS
DDFFM_1	−0.5232	0.3188	25.49	0.9066	0.0540
DDFFM_2	0.8327	0.2760			
DDFFM_3	0.3032	0.8632			

## Data Availability

The datasets used in this study are publicly available: StateHaze1k, https://www.dropbox.com/scl/fi/wtga5ltw5vby5x7trnp0p/Haze1k.zip?rlkey=70s52w3flhtif020nx250jru3&e=1&dl=0 (accessed on 16 August 2025); RICE, https://drive.google.com/file/d/1CricZtIj28BGFvkD_x-W8fSexPiDtgHk/view (accessed on 16 August 2025); RRSHID, https://drive.google.com/file/d/1uBwHM8tyw69xafFHd01vMERs3TadUmxT/view (accessed on 16 August 2025). The source code implementing DFFNet is available at https://github.com/chen29181/DFFNet (accessed on 16 August 2025). Implementations of comparison methods are not included in our repository; we relied on their official releases as cited.
